# Parental origin of chromosomes influences crossover activity within the *Kcnq1 *transcriptionally imprinted domain of *Mus musculus*

**DOI:** 10.1186/1471-2199-10-43

**Published:** 2009-05-13

**Authors:** Siemon H Ng, Rose Madeira, Emil D Parvanov, Lorin M Petros, Petko M Petkov, Kenneth Paigen

**Affiliations:** 1 Center for Genome Dynamics, The Jackson Laboratory, 600 Main Street, Bar Harbor, Maine, USA

## Abstract

**Background:**

Among the three functions of DNA, mammalian replication and transcription can be subject to epigenetic imprinting specified by the parental origin of chromosomes, and although there is suggestive indication that this is also true for meiotic recombination, no definitive evidence has yet been reported.

**Results:**

We have now obtained such evidence on mouse chromosome 7 by assaying meiotic recombination as it occurs in reciprocal F1 mice. A 166 kb region near the *Kcnq1 *transcriptionally imprinted domain showed significantly higher recombination activity in the CAST×B6 parental direction (p < 0.03). Characterizing hotspots within this domain revealed a cluster of three hotspots lying within a 100 kb span, among these hotspots, *Slc22a18 *showed a definitive parent of origin effect on recombination frequency (p < 0.02). Comparing recombination activity in the mouse *Kcnq1 *and neighboring *H19-Igf2 *imprinted domains with their human counterparts, we found that elevated recombination activity in these domains is a consequence of their chromosomal position relative to the telomere and not an intrinsic characteristic of transcriptionally imprinted domains as has been previously suggested.

**Conclusion:**

Similar to replication and transcription, we demonstrate that meiotic recombination can be subjected to epigenetic imprinting and hotspot activity can be influenced by the parental origin of chromosomes. Furthermore, transcriptionally imprinted regions exhibiting elevated recombination activity are likely a consequence of their chromosomal location rather than their transcriptional characteristic.

## Background

DNA serves three major functions; it is replicated, providing the material for hereditary transmission from one generation to the next; it is transcribed, expressing its stored information as a variety of RNA products; and it undergoes meiotic recombination, generating population variation and substrates for evolution. Two of these processes, replication [1,2] and transcription [3], can be subject to parentally determined epigenetic modification, generally known as genomic imprinting, and recombination has also been postulated to be affected by imprinting [4-6].

Transcriptional imprinting is characterized by the silencing of one parental-specific allele in the offspring [7-9], and asynchronous DNA replication due to the parental origin of chromosomes has been observed in some transcriptionally imprinted domains [1,2,10]. While the mechanisms of genomic imprinting and its epigenetic regulation of gene expression and replication have been extensively studied (for a review see [3]), a direct demonstration of epigenetic controls in meiotic recombination has not been available.

Among the three functions of DNA, meiotic recombination is the only process that involves physical interactions between the two parental chromosomes. Meiotic recombination begins in meiosis I with a Spo11 mediated double strand break (DSB) [11]. This break is subsequently repaired via either the double strand break repair pathway, where DNA sequences flanking the DSB are exchanged between non-sister chromatids resulting in a crossover, or the synthesis-dependent strand annealing pathway in which the DSB is repaired using a non-sister chromatid as the copying template generating a short region of gene conversion without the exchange of flanking sequences (a non-crossover). For a review of the recombination pathways see [11,12].

In mammals, where recombination initiates prior to synapsis, meiotic crossovers occur at preferred 1–2 kb regions, known as recombination hotspots. Currently, no comprehensive model exists that accounts for the location and activity of recombination hotspots. Regional differences in recombination frequencies have been associated with both transcriptionally imprinted regions and regions near telomeres. Telomeric regions in both humans and mice generally have higher male than female recombination activity [13-15] and imprinted regions located near telomeres, such as the human *H19-Igf2 *and *Kcnq1 *domains, show higher male recombination activity [16].

The mouse *H19-Igf2 *and *Kcnq1 *imprinted domains, located on the distal region of chromosome 7, provide a model for examining if meiotic recombination hotspot can be subjected to epigenetic imprinting where recombination activity is affected by the parental origin of the recombining chromatids. Mapping recombination rates in this region, we found one region near the *Kcnq1 *transcriptionally imprinted domain subject to meiotic recombination imprinting as evidenced by recombination being much elevated in F1 animals arising from one parental direction of the cross versus the opposite. Fine mapping of recombination activity within this region to hotspot level resolution showed a cluster of three hotspots whose meiotic recombination activities are imprinted.

Additionally, while the imprinting mechanisms are identical in the *H19-Igf2 *and *Kcnq1 *imprinted domains in humans and mice [17,18], the genomic arrangement of these two domains relative to the telomere is inverted in the mouse genome. Our analysis of this region showed that in mice recombination activity is elevated only in the *Kcnq1 *domain, suggesting that recombination activities of hotspots within transcriptionally imprinted domains are influenced by their chromosomal position and not all transcriptionally imprinted domains exhibit elevated recombination activity.

## Results

### High recombination was observed within the *Kcnq1 *imprinted domain but not within *H19-Igf2 *imprinted domain

**Figure 1:**
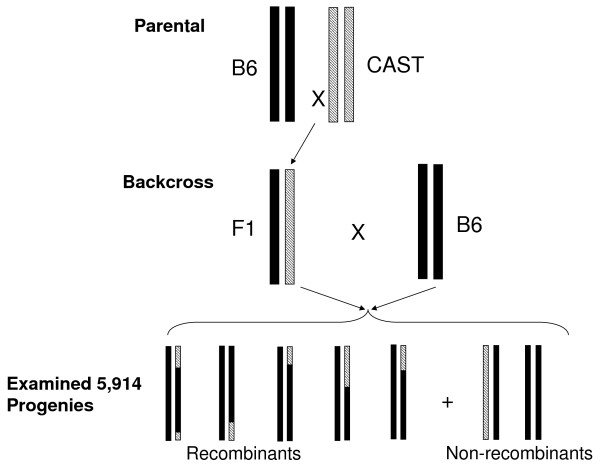
**Schema of the mouse crosses**. C57B6/J (B6) were crossed with CAST/EiJ to obtain heterozygous F1 generation. Recombination activities in the F1 generation were monitored by backcrossing these with B6 and examining their progenies.

**Figure 2:**
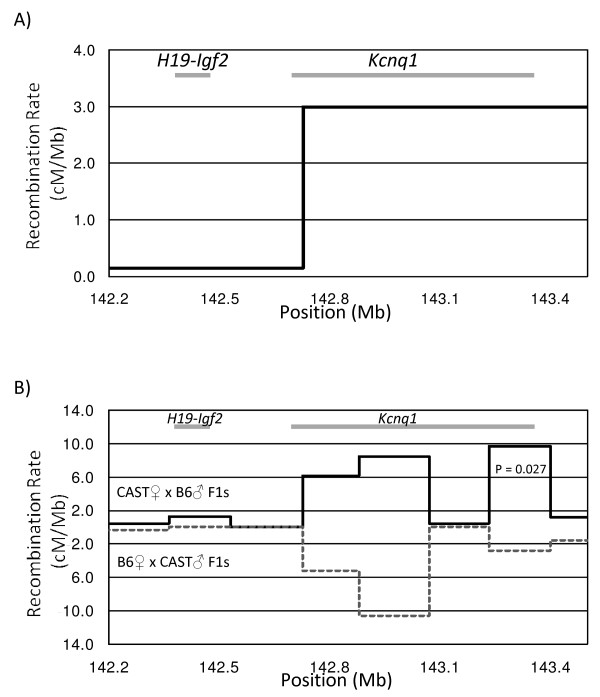
**Recombination map for the *H19-Igf2 *and *Kcnq1 *region of mouse chromosome 7**. (A) Coarse recombination mapping across the two transcriptionally imprinted domains (labeled and highlighted by grey bars). Average crossover rate for *H19-Igf2 *region was 0.14 cM/Mb while the Kcnq1 domain showed a recombination frequency of 2.98 cM/Mb. (B) Comparison of recombination activity between CAST♀xB6♂ and B6♀xCAST♂ parental crosses at an average 172 kb resolution. Only one interval showed significant difference in recombination activity between the reciprocal parental cross (P-value = 0.027, corrected for multiple testing by the Bioufferoni technique).

We mapped crossover events within the *H19-Igf2 *and *Kcnq1 *transcriptionally imprinted domains on mouse chromosome 7 (142.366 – 143.400 Mb, NCBI Build 36) in 5,914 meioses of a cross between the C57/BL6J and CAST/EiJ strains (Figure 1). Only three crossovers were observed within the *H19-Igf2 *domain (142.366 – 142.727 Mb), yielding a sex-averaged recombination rate of 0.14 cM/Mb, approximately 4 times lower than the genome average of 0.5 to 0.6 cM/Mb [6,15,19] (Figure 2A). In contrast, the *Kcnq1 *imprinted domain (142.727–143.400 Mb), encompassing genes between *Th *and *Nap1l4*, contained 119 recombinants yielding a crossover activity of 2.98 cM/Mb, five times the mouse genome-wide average.

We further mapped the recombination activity within this latter region to an average resolution of about 172 kb and compared the recombination activity between reciprocal parental crosses (B6♀ × CAST♂ vs. CAST♀ × B6♂) to detect if the chromosomal origin of the parental chromosome affects meiotic recombination. A meiotic recombination imprinting effect was found in a 166 kb region near distal side of the *Kcnq1 *transcriptionally imprinted domain (Figure 2B). This region contained a total of 31 recombinants with 7 recombinants arising in B6 × CAST F1 mice and 24 recombinants arising in CAST × B6 F1 mice (P_FET _= 0.027 after Bonferroni correction for multiple testing). None of the other intervals within the *H19-Igf2 *and *Kcnq1 *transcriptionally imprinted domains showed any significant differences in recombination activity between the two reciprocal parental crosses.

### Fine mapping revealed five novel recombination hotspots within the *Kcnq1 *transcriptionally imprinted domain

**Figure 3:**
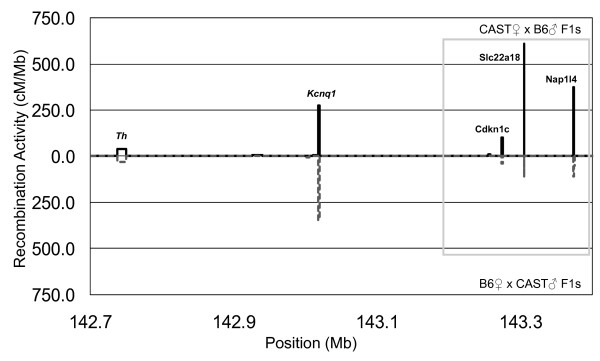
**Meiotic recombination imprinting at three hotspots within the Kcnq1 domain**. Recombination events within the Kcnq1 domain were mapped at hotspot resolution. Hotspot frequencies from reciprocal parental crosses were compared at five novel hotspots. Hotspots *Th *and *Kcnq1 *were not affected by parental origin of chromosome. Hotspots *Cdkn1c*, *Slc22a18 *and *Nap1l4 *(boxed) clustered within a 100 kb region and showed meiotic recombination imprinting with higher crossover activity in the CAST♀ × B6♂ direction of the parental cross.

To better understand whether meiotic imprinting influences recombination at a regional or hotspot level, we mapped the crossovers occurring in the *Kcnq1 *domain to hotspot resolution, revealing five highly active hotspots. These hotspots were named according to their closest neighboring gene (Figure 3, Table 1). Hotspot *Th *was mapped to an 11 kb interval (the limit of resolution possible with available SNPs), while hotspots *Kcnq1*, *Cdkn1c*, *Slc22a18 *and *Nap1l4 *were mapped to a resolution of 2.3 kb or less. We considered the 11 kb *Th *interval as a single hotspot as it is unlikely that multiple hotspots will be found within such a short interval [6]. Of the five hotspots, three (*Th*, *Cdkn1c *and *Nap114*) were located in intergenic regions and two hotspots were found within introns; hotspot *Kcnq1 *within a large intron of 65 kb while hotspot *Slc22a18 *is located within a short 4 kb intron.

**Table 1:** Location and flanking sequences of the five hotspots within the *Kcnq1 *transcriptionally imprinted domain.

Hotspot	Location (Mb – Build 36)	Left Flanking Sequence (5'-3')	Right Flanking Sequence (5'-3')
*Th*	142.7379–142.7497	CAGCTTCAGGTCTACTTTGGT	CTAGAAAGAAACCCAGTACAC
*Kcnq1*	143.0172–143.0196	CTAAGTAGCAAACAATGCAA	TTTGAAACACATGGAAGGCAC
*Cdkn1c*	143.2733–143.2749	AGCTAAGTCAGTTTAGTTTCC	TGTTGGTGCTAGCAGGACACA
*Slc22a18*	143.3044–143.3050	GGTTAGGGTCAGGGATGTGAG	TCTTTGGGCCCACACACTTCC
*Nap1l4*	143.3737–143.3749	GGCTAGCTCCTCCATAGCCAC	GACAGCCACCACAGGTAACCC

The interval displaying meiotic recombination imprinting encompassed hotspots *Cdkn1c*, *Slc22a18 *and *Nap1l4*. These hotspots clustered within a 100 kb region (NCBI build 36, 143.27 – 143.37 Mb), and, as a cluster, all three hotspots showed elevated recombination activity in CAST♀ × B6♂ F1 mice regardless of whether the recombination occurs in the male or female germline (P_FET _= 0.005; the number of recombinants is slightly less than those detected at the regional level as some crossover events are not associated with any particular hotspot). This differential recombination activity between reciprocal crosses was statistically significant for *Slc22a18 *(P_FET _= 0.02 without Bonferroni correction and P_FET _= 0.10 with Bonferroni correction), however, the number of crossovers at each of flanking hotspots, although consistent with a parent of origin effect, was not sufficient to provide low *p *values (Table 2). Individually, hotspot *Cdkn1c *and *Nap1l4 *showed similar rate of recombination when the recombination event occurs in the female F1 animals, however, this is likely due to the limited number of crossovers detected at these flanking hotspots.

**Table 2:** Epigenetic imprinting influences recombination activity within the *Kcnq1 *imprinted domain.

	Sex-Averaged Recombination Activity		Number of Crossovers in B6♀ × CAST♂ Parental Cross			Number of Crossovers in CAST♀ × B6♂ Parental Cross			Sex-averaged CxB:BxC Ratio			
Hotspot	cM	cM/Mb	Male	Female	Total	Male	Female	Total	Ratio		P_FET _Value	

*Th*	0.40	33.7	9	2	11	11	2	13	1.2	0.92	0.83	0.63
*Kcnq1*	0.73	308	25	2	27	19	3	22	0.81		0.47	
*Cdkn1c*	0.12	71.2	0	2	2	3	2	5	2.5	3.3	0.45	0.005
*Slc22a18*	0.22	367	2	0	2	9	2	11	5.5		0.02	
*Nap1l4*	0.15	243	3	0	3	7	0	7	2.3		0.34	

Hotspots *Slc22a18 *and its flanking hotspots, *Cdkn1c *and *Nap1l4*, clustered within 100 kb and showed a regionally significant increase in crossover activity in F1 animals from CAST♀ × B6♂ parents. Both sexes showed higher number of recombinant in the CAST♀ × B6♂ parental cross within the 100 kb cluster with a C×B:B×C ratio of 3.8 for males and 2.5 for females.

### Meiotic recombination imprinting and possible chromatid of origin effects

One possible site of action for meiotic recombination imprinting is the choice of the chromatid on which the initiating DSB occurs. When DSBs are initiated on one chromatid, the subsequent repair processes use the homologous chromatid as a template, resulting in conversion of short sequence stretch at the center of the hotspot to the donor sequence [20]. This makes it possible to determine the chromatid on which the initiating double strand break occurs when informative SNPs across the hotspot are available. For hotspot *Kcnq1*, which is not recombinationally imprinted, the initiating chromatid showed no evidence of parental imprinting. However, at hotspot *Cdkn1c*, which is recombinationally imprinted, the limited number of crossovers suggests a possible initiation bias on the paternal chromosome.

At hotspot *Kcnq1*, the molecular details of crossing over were typed among 49 offspring using three additional SNP markers. Among progeny of both reciprocal parents, the central marker was three times more likely to be of CAST genotype in both parental directions suggesting that DSB initiation occurs preferentially on the B6 chromosome regardless of its parental origin; thus, no meiotic recombination imprinting was detectable at this hotspot in our study (Table 3 and 4). Among the hotspots where meiotic recombination imprinting was detected, only *Cdkn1c *contained informative SNPs spanning across the region of crossover exchange, and the seven recombinants at this hotspot were typed for these internal SNPs (Table 3 and 5). Limited by the small number of crossovers occurring at this hotspot, we found only suggestive confirmation that direction of the parental cross influenced the choice of the initiating chromosome with DSBs preferentially initiating on the paternal chromosome (P-value < 0.13, Table 3).

**Table 3:** DSB initiation at hotspot *Kcnq1 *is unaffected by epigenetic imprinting while hotspot *Cdkn1c *showed suggestive bias of DSB initiation.

Initiating Parental Chromosome	*Kcnq1*	*Cdkn1c*
Paternal	23	6
Maternal	26	1
P-value_(FET)_	0.77	0.13

Examining 49 recombinant offspring at hotspot Kcnq1, which is not recombinationally imprinted, showed DSBs can occur on both the paternal and maternal. However, at hotspot *Cdkn1c*, a recombinationally imprinted hotspot, 6 of the 7 recombinants were initiated on the paternal chromosome giving suggestive evidence that DSBs is also subjected to meiotic imprinting (p < 0.13).

**Table 4:** DSB initiation at hotspot *Kcnq1 *is unaffected by epigenetic imprinting.

	Genotypes				Initiating Chromosome		
Animals	SNP1 (143,017,421)	SNP2 (143,018,553)	SNP3 (143,019,622)	Count	Parental		Strain

B6×CAST	B	C	C	10	Maternal	21	B6
	C	C	B	11			
	
	B	B	C	4	Paternal	6	CAST
	C	B	B	2			

CAST×B6	B	B	C	3	Maternal	5	CAST
	C	B	B	2			
	
	B	C	C	8	Paternal	17	B6
	C	C	B	9			

Examining 49 recombinant offspring at the *Kcnq1 *hotspot showed DSBs preferentially occurs on the B6 chromosome, regardless of the chromosome's parental origin. SNP position indicated in parentheses (base pairs – Build 36).

**Table 5:** Genotyping details for the crossover recombinants at hotspot *Cdkn1c*.

Animal	Initiating Parental Chromosome	SNP1 (143,273,317)	SNP2 (143,273,451)	SNP3 (143,273,907)	SNP4 (143,274,027)	SNP5 (143,274,103)	SNP6 (143,274,837)	SNP7 (143,274,943)
B6×CAST	Paternal	B	B	B	B	**B**	**C**	C
B6×CAST	Paternal	B	B	B	B	**B**	**C**	C
CAST×B6	Paternal	C	C	C	C	**C**	**B**	B
CAST×B6	Paternal	B	**B**	**C**	C	C	C	C
CAST×B6	Paternal	B	**B**	**C**	C	C	C	C
CAST×B6	Paternal	C	C	C	C	**C**	**B**	B
CAST×B6	Maternal	B	B	B	B	**B**	**C**	C

Seven SNPs were used to fine map hotspot *Cdkn1c*. The center of the hotspot was mapped to between SNP3 and SNP5. The central SNP (SNP4) is converted to the non-initiating strand during meiotic recombination. A slight statistical bias is found for DSB initiation on the paternal chromosome (P = 0.13). SNP positions are indicated in parentheses (base pairs – Build 36).

## Discussion

### Parental origin of chromosomes can influence the recombination activity at specific hotspots

When recombination was mapped along the entirety of mouse chromosome 1, a significant excess of recombination intervals was observed in which preferential recombination occurred in one parental direction of the cross or the other, but no single interval showed a statistically significant preference by itself [6].

Mapping the recombination activity on chromosome 7 at a regional scale, we found an interval showing definitive evidence of meiotic recombination imprinting. Fine mapping revealed meiotic recombination imprinting influences at the hotspot level involving hotspots *Cdkn1c*, *Slc22a18 *and *Nap1l4*. Their close proximity, clustering within a 100 kb region, suggests that the parental origin of chromosomes exerts a regional influence on meiotic recombination activity (Figure 3). While these hotspots with imprinted meiotic recombination activity are located in a transcriptionally imprinted region, it will require additional examples of recombination imprinting to clarify the extent to which meiotic recombination imprinting overlaps with and/or is related to transcriptional imprinting. It is quite possible that some hotspots with imprinted meiotic recombination activity are not located within a transcriptionally imprinted domain. Another possibility, though remote, is that an imprinted trans-acting factor controls the recombination activities of hotspots showing parent of origin effects.

While DNA methylation is a well studied epigenetic marker controlling transcription of imprinted genes, the methylation pattern is reportedly erased and reestablished prior to the beginning of meiosis in male [21-23] but erased and not yet established prior to meiotic recombination in females [24]. The observed recombination activity imprinted in the CAST♀ × B6♂ parental direction is unlikely to be a consequence of DNA methylation, and another imprinting epigenetic marker may be responsible for differentiating the two parental chromosomes [25,26]. In considering possible mechanisms of meiotic recombination imprinting, it is also worth noting that it is only during recombination, not replication or transcription, that homologous chromosomes come into physical contact with each other. For transcriptional imprinting and replication imprinting there is no indication of a sex bias where the imprinted pattern is influenced by the sex of an individual, and similarly we do did not find sex to influence meiotic recombination imprinting.

### DSB initiation may be influenced by epigenetic imprinting

If DSB initiation occurs on both parental chromosomes at equal frequencies, the number of DSBs will be constant regardless of the parental origin of chromosomes, and no influence on the overall recombination activity would be observed. For example, if the DSB initiating recombination occurs preferentially on the B6 chromatid but epigenetic imprinting does not play a role, one would expect equal rates of recombination in B6♀ × CAST♂ and CAST♀ × B6♂ F1 mice. Conversely, if the DSB is equally likely to occur on a B6 or CAST chromatid and epigenetic imprinting does occur, suppressing DSB formation on the maternal chromosome, the decrease in DSBs would affect both the maternal B6 chromatid in B6♀ × CAST♂ mice and the maternal CAST chromatid in CAST♀ × B6♂ mice equally and no change in recombination frequency would be observed. Thus, detection of meiotic recombination imprinting requires both a strong preference for recombination to initiate on one specific parental chromatid and that this region of the genome is subject to epigenetic imprinting. In this latter case, if DSBs initiate preferentially on the B6 chromatid (indeed, such preference has been previously observed in human and mouse [6,27]) and epigenetic imprinting suppresses DSB initiation on the maternal chromatid, one would observed a decrease in recombination activity in the B6♀ × CAST♂ mice as the number of DSB initiation events on the B6 maternal chromatid (and the subsequent recombination activity) is reduced. In contrast, the number of recombination events will remain unchanged in the CAST♀ × B6♂ mice where the maternal CAST chromatid is suppressed but DSB initiation on the paternal B6 chromatid in unaffected.

Although our data is limited by the number of recombinants observed at hotspot *Cdkn1c*, even with 5,914 meioses, they do lean in the direction of suggesting that meiotic recombination imprinting regulates recombination activity by directing DSBs preferentially towards one chromatid. It is possible that DSB initiation is favored towards the paternal chromosome at all of the imprinted hotspots (*Cdkn1c*, *Slc22a18 *and *Nap1l4*) clustered within 100 kb; unfortunately the lack of suitable SNPs precluded testing the remaining hotspots within this region. This is the first detailed examination of meiotic recombination imprinting at a specific hotspot.

We should also point out two alternative theoretical possibilities that, although unlikely, could explain our results. There could exist a trans-acting gene that is both subject to imprinting and whose B6 and CAST alleles differ in their ability to control recombination in the *Slc22a18 *region. This would require imprinting of gene function during meiosis, shifting the imprinted region from *Slc22a18 *to another site. It is also possible that a trans-acting gene controlling recombination in the *Slc22a18 *region is located on the X chromosome as reciprocal F1 males are X^B6^Y^CAST ^v. X^CAST^Y^B6^; again, the B6 and CAST alleles of this putative gene would have to differ in their activating ability. In this context, we should point out that trans control of some hotspot activities, albeit by an autosomal locus, has been reported [28,29].

We have expanded our analysis to over 10 Mb near the distal end of chromosome 7, but no other region showed a meiotic recombination imprinting effect bias similar to those observed near the *Kcnq1 *domain (data not shown). It is likely that meiotic imprinted hotspots are not a common occurrence, and the challenge is now to find additional hotspots subject to meiotic recombination imprinting and characterize their molecular mechanism.

### Overall recombination activity in the *H19-Igf2 *and *Kcnq1 *imprinted domains is dependent upon genomic positioning and not transcriptional imprinting

Human transcriptionally imprinted regions have been associated with elevated recombination frequencies [4,5,16,30]. In humans, both the *H19-Igf2 *and *Kcnq1 *transcriptionally imprinted domains showed elevated male recombination activities [16]. The low crossover activity at the mouse *H19-Igf2 *region is in marked contrast to the human *H19-Igf2 *recombination pattern. In addition, the difference in recombination activity between the two transcriptionally imprinted domains in mice indicates that elevated recombination is not necessarily correlated with transcriptional imprinting.

It is likely that the location of hotspots relative to the telomeres has a greater influence on recombination activity. It appears that sex-biased recombination activity (or any recombination activity) is not a general characteristic of mammalian transcriptionally imprinted domains, and recombination within these imprinted regions is likely influenced by their relative position to the telomeres.

## Conclusion

We mapped five new recombination hotspots in the *Kcnq1 *imprinted domain, and comparing recombination activity of F1 animals from reciprocal parents obtained evidence that meiotic recombination imprinting can influence crossover activity at a cluster of three closely spaced hotspots. These results suggest that, like replication and transcription, meiotic recombination can be subjected to epigenetic imprinting. However, any epigenetic marker for recombination is likely independent of DNA methylation as the methylation pattern of germ cells are reported to be erased and re-established prior to meiosis. Recombination activity in the *H19-Igf2 *and *Kcnq1 *chromosomal regions showed that transcriptionally imprinted mammalian regions do not necessarily have elevated recombination activity. The higher recombination activity observed at *Kcnq1 *is likely a consequence of their chromosomal location rather than their imprinted characteristic.

## Methods

### Mouse Strain and Spleen DNA extraction

Mice were purchased from The Jackson Laboratory. The DNA samples used in this study were previously described elsewhere [6]. They came from a total of 5,914 offspring of C57BL/6J (B6) × CAST/EiJ (CAST) F1 mice backcrossed with B6. DNA for genotyping was obtained from spleen samples from 2,917 offspring of B6♀ × CAST♂F1 and 2,997 offspring of CAST♀ × B6♂F1. Briefly, mouse spleens were digested overnight in 900 μl buffer containing 50 mM KCl, 10 mM Tris-HCl (pH 8.3), 2.5 mM MgCl_2_, 0.1 mg/ml gelatin, 0.45% v/v Nonidet P40, 0.45% v/v Tween 20, and 60 mg/ml proteinase K. After digestion, 100 μl of 100 mM Tris-HCl (pH 8.0) were added and samples were diluted 10× in 10 mM Tris-HCl (pH 8.0) for genotyping.

### Broad-scale 200 kb Genotyping

All progeny were initially genotyped using two SNP markers flanking the H19-Igf2 and Kcnq1 transcriptionally imprinted regions. Crossovers were detected as a transition from homozygous to heterozygous genotype or vice versa. Informative meioses were further mapped using 4 additional SNP markers to achieve a resolution of less than 200 kb. This broad-scale genotyping was carried out by KBiosciences (UK) using SNPs markers from the publicly available Perlegen SNP database [see additional file 1].

### Hotspot Resolution SNP Genotyping

To fine map hotpots to less than 5 kb resolution, SNPs were obtained from the Perlegen SNP database. For hotspots *Kcnq1*, *CdKn1c*, *Slc22a18 *and *Nap1l4*, where the Perlegen data was incomplete, additional SNPs were obtained by sequencing CAST and B6 genomic DNA. New assays were developed for the Chemicon Amplifluor SNPs HT FAM-JOE System (Millipore, Billerica, MA). Primers were designed using the Amplifluor AssayArchitect software . Reactions were carried out in 384 well plates using 5 μl reaction volume consisting of 30 ng DNA, 0.5 μl of 10× Reaction Mix S Plus, 0.4 μl 2.5 mM each dNTP, 0.25 μl 20× FAM Primer, 0.25 μl 20× JOE Primer, 0.25 μl SNP specific primer mix (0.5 μM green tailed allele-specific primer, 0.5 μM red tailed complementary allele-specific primer, 7.5 μM solution common reverse primer) and 0.025 μl Titanium Taq DNA polymerase (Clontech, Mountain View, CA). PCR was performed as suggested by the manufacturer. End-point discrimination of the alleles was carried out on an ABI 7900 HT Real-time PCR system (Applied Biosystems, Framingham, MA).

## Authors' contributions

SN, PP and KP conceived of the study and participated in the design of the experiments. LP, EP participated in the design and optimization of the study. SN carried out the primer design, SNP genotyping, analysis of the data, and drafted the manuscript. RM carried out the high resolution SNP genotyping and primer design.

## Supplementary Material

Additional file 1 **SNPs markers used in the study for mapping recombination events**. The two tables provided represent the SNPs used in the study for mapping recombination events (Table 1) and the additional SNP markers found by sequencing used for in higher resolution mapping of the recombination hotspots (Table 2). Click here for file

## References

[B1] Izumikawa Y, Naritomi K, Hirayama K (1991). Replication asynchrony between homologs 15q11.2: cytogenetic evidence for genomic imprinting. Human genetics.

[B2] Kagotani K, Takebayashi S, Kohda A, Taguchi H, Paulsen M, Walter J, Reik W, Okumura K (2002). Replication timing properties within the mouse distal chromosome 7 imprinting cluster. Bioscience, biotechnology, and biochemistry.

[B3] Edwards CA, Ferguson-Smith AC (2007). Mechanisms regulating imprinted genes in clusters. Current opinion in cell biology.

[B4] Lercher MJ, Hurst LD (2003). Imprinted chromosomal regions of the human genome have unusually high recombination rates. Genetics.

[B5] Robinson WP, Lalande M (1995). Sex-specific meiotic recombination in the Prader – Willi/Angelman syndrome imprinted region. Human molecular genetics.

[B6] Paigen K, Szatkiewicz JP, Sawyer K, Leahy N, Parvanov ED, Ng SH, Graber JH, Broman KW, Petkov PM (2008). The recombinational anatomy of a mouse chromosome. PLoS Genet.

[B7] Barton SC, Surani MA, Norris ML (1984). Role of paternal and maternal genomes in mouse development. Nature.

[B8] Monk M (1987). Genomic imprinting. Memories of mother and father. Nature.

[B9] Surani MA, Barton SC, Norris ML (1984). Development of reconstituted mouse eggs suggests imprinting of the genome during gametogenesis. Nature.

[B10] Knoll JH, Cheng SD, Lalande M (1994). Allele specificity of DNA replication timing in the Angelman/Prader-Willi syndrome imprinted chromosomal region. Nature genetics.

[B11] Neale MJ, Keeney S (2006). Clarifying the mechanics of DNA strand exchange in meiotic recombination. Nature.

[B12] Arnheim N, Calabrese P, Tiemann-Boege I (2007). Mammalian meiotic recombination hot spots. Annual review of genetics.

[B13] Lynn A, Kashuk C, Petersen MB, Bailey JA, Cox DR, Antonarakis SE, Chakravarti A (2000). Patterns of meiotic recombination on the long arm of human chromosome 21. Genome research.

[B14] Myers S, Bottolo L, Freeman C, McVean G, Donnelly P (2005). A fine-scale map of recombination rates and hotspots across the human genome. Science.

[B15] Shifman S, Bell JT, Copley RR, Taylor MS, Williams RW, Mott R, Flint J (2006). A high-resolution single nucleotide polymorphism genetic map of the mouse genome. PLoS biology.

[B16] Sandovici I, Kassovska-Bratinova S, Vaughan JE, Stewart R, Leppert M, Sapienza C (2006). Human imprinted chromosomal regions are historical hot-spots of recombination. PLoS Genet.

[B17] Kaffer CR, Grinberg A, Pfeifer K (2001). Regulatory mechanisms at the mouse Igf2/H19 locus. Molecular and cellular biology.

[B18] Umlauf D, Goto Y, Cao R, Cerqueira F, Wagschal A, Zhang Y, Feil R (2004). Imprinting along the Kcnq1 domain on mouse chromosome 7 involves repressive histone methylation and recruitment of Polycomb group complexes. Nature genetics.

[B19] Dietrich WF, Miller J, Steen R, Merchant MA, Damron-Boles D, Husain Z, Dredge R, Daly MJ, Ingalls KA, O'Connor TJ (1996). A comprehensive genetic map of the mouse genome. Nature.

[B20] Baudat F, de Massy B (2007). Cis- and trans-acting elements regulate the mouse Psmb9 meiotic recombination hotspot. PLoS Genet.

[B21] Feil R, Walter J, Allen ND, Reik W (1994). Developmental control of allelic methylation in the imprinted mouse Igf2 and H19 genes. Development (Cambridge, England).

[B22] Lucifero D, Mertineit C, Clarke HJ, Bestor TH, Trasler JM (2002). Methylation dynamics of imprinted genes in mouse germ cells. Genomics.

[B23] Oakes CC, La Salle S, Smiraglia DJ, Robaire B, Trasler JM (2007). Developmental acquisition of genome-wide DNA methylation occurs prior to meiosis in male germ cells. Developmental biology.

[B24] Bourc'his D, Proudhon C (2008). Sexual dimorphism in parental imprint ontogeny and contribution to embryonic development. Mol Cell Endocrinol.

[B25] Seki Y, Hayashi K, Itoh K, Mizugaki M, Saitou M, Matsui Y (2005). Extensive and orderly reprogramming of genome-wide chromatin modifications associated with specification and early development of germ cells in mice. Developmental biology.

[B26] Seki Y, Yamaji M, Yabuta Y, Sano M, Shigeta M, Matsui Y, Saga Y, Tachibana M, Shinkai Y, Saitou M (2007). Cellular dynamics associated with the genome-wide epigenetic reprogramming in migrating primordial germ cells in mice. Development.

[B27] Jeffreys AJ, Neumann R (2005). Factors influencing recombination frequency and distribution in a human meiotic crossover hotspot. Human molecular genetics.

[B28] Grey C, Baudat F, de Massy B (2009). Genome-wide control of the distribution of meiotic recombination. PLoS biology.

[B29] Parvanov ED, Ng SH, Petkov PM, Paigen K (2009). Trans-regulation of mouse meiotic recombination hotspots by Rcr1. PLoS biology.

[B30] Paldi A, Gyapay G, Jami J (1995). Imprinted chromosomal regions of the human genome display sex-specific meiotic recombination frequencies. Curr Biol.

