# Supplementary material for: Parental origin of chromosomes influences crossover activity within the Kcnq1 transcriptionally imprinted domain of Mus musculus
Source: BMC Mol Biol. 2009 May 13;10:43. doi: 10.1186/1471-2199-10-43 (PMC2689222; doi:10.1186/1471-2199-10-43)
Supplement: Additional file 1 — SNPs markers used in the study for mapping recombination events. The two tables provided represent the SNPs used in the study for mapping recombination events (Table 1) and the additional SNP markers found by sequencing used for in higher resolution mapping of the recombination hotspots (Table 2). [file 1471-2199-10-43-S1.doc]

| **Position (Build 36)** | **SNP ID** | **SNP Polymorphism (B6/CAST)** |
| --- | --- | --- |
| 142365500 | NES11525910 | T/G |
| 142531877 | NES11519771 | T/C |
| 142609591 | NES11540049 | T/C |
| 142632069 | NES11539520 | A/T |
| 142696985 | NES11537316 | T/A |
| 142727021 | NES11536175 | C/T |
| 142737911 | NES11535790 | A/G |
| 142749696 | NES11535081 | C/A |
| 142754653 | NES11534933 | G/T |
| 142755898 | NES11534836 | C/T |
| 142761808 | NES11534444 | A/T |
| 142763694 | NES11534222 | G/A |
| 142773377 | NES11533679 | C/G |
| 142826955 | NES11531529 | T/C |
| 142842933 | NES11530574 | A/G |
| 142880812 | NES11529300 | A/G |
| 142925954 | NES11527499 | T/C |
| 142940507 | NES11526709 | C/A |
| 142953433 | NES11526163 | T/A |
| 142972978 | NES11525561 | A/T |
| 142993337 | NES11524709 | A/C |
| 143001847 | NES11524231 | C/T |
| 143005531 | NES11524252 | C/A |
| 143008891 | NES11524105 | C/T |
| 143010847 | NES11523889 | T/C |
| 143017262 | NES11523711 | A/G |
| 143019622 | NES11523540 | T/G |
| 143071413 | NES11522147 | G/T |
| 143234126 | NES11516343 | A/C |
| 143254445 | NES11530947 | G/A |
| 143273317 | See Supplementary Table 2 | |
| 143273451 | See Supplementary Table 2 | |
| 143273907 | See Supplementary Table 2 | |
| 143274027 | See Supplementary Table 2 | |
| 143274103 | See Supplementary Table 2 | |
| 143274837 | See Supplementary Table 2 | |
| 143274943 | See Supplementary Table 2 | |
| 143276197 | NES11529804 | A/G |
| 143285240 | NES11529517 | C/T |
| 143297062 | NES11528886 | C/T |
| 143303254 | NES11528829 | A/G |
| 143303695 | NES11528624 | T/C |
| 143303959 | See Supplementary Table 2 | |
| 143304188 | See Supplementary Table 2 | |
| 143304371 | See Supplementary Table 2 | |
| 143304455 | See Supplementary Table 2 | |
| 143305040 | NES11528601 | T/C |
| 143306395 | NES11528546 | T/C |
| 143306694 | NES11528547 | C/T |
| 143336406 | NES11527181 | G/T |
| 143346651 | NES11526600 | T/C |
| 143368357 | NES11525449 | C/T |
| 143373210 | NES11525195 | T/C |
| 143373324 | See Supplementary Table 3 | |
| 143373708 | See Supplementary Table 3 | |
| 143374970 | NES11525203 | A/G |
| 143377814 | NES11525012 | C/T |
| 143378207 | NES11525014 | A/C |
| 143400032 | NES11523629 | A/G |

Supplemental Table 1: List of SNPs used for genotyping across the 1.1 Mb distal region of mouse chromosome 7.

| Hotspot | SNP Location  (Mb – Build 36) | Polymorphism  (B6 / CAST) | Amplifluor SNP Assay Oligos (5’ – 3’) | |
| --- | --- | --- | --- | --- |
| *Cdkn1c* | 143,273,317 (SNP 1) | A/G | Allele 1 | GAAGGTCGGAGTCAACGGATTCCTGGAGCAGCTAAGTCAGTTT |
|  |  |  | Allele 2 | GAAGGTGACCAAGTTCATGCTTCCTGGAGCAGCTAAGTCAGTTC |
|  |  |  | Reverse | CTATGCGGGTTGTTGTGAGT |
|  | 143,273,451 (SNP 2) | T/C | Allele 1 | GAAGGTGACCAAGTTCATGCTCAGAACAGAGTCCGAGTCC |
|  |  |  | Allele 2 | GAAGGTCGGAGTCAACGGATTCCAGAACAGAGTCCGAGTCT |
|  |  |  | Reverse | AGCCAAGAAACTGGTGATGTT |
|  | 143,273,907 (SNP 3) | T/C | Allele 1 | GAAGGTGACCAAGTTCATGCTGGGGTCTCTTCATATGGGC |
|  |  |  | Allele 2 | GAAGGTCGGAGTCAACGGATTTTCTGGGGTCTCTTCATATGGGT |
|  |  |  | Reverse | GGCACAAGATGGAGACACTTT |
|  | 143,274,027 (SNP 4) | C/A | Allele 1 | GAAGGTCGGAGTCAACGGATTCTGTTCTCTCTCTCCAGGGTT |
|  |  |  | Allele 2 | GAAGGTGACCAAGTTCATGCTGTTCTCTCTCTCCAGGGTG |
|  |  |  | Reverse | GTCTCCATCTTGTGCCTACTGT |
|  | 143,274,103 (SNP 5) | A/C | Allele 1 | GAAGGTCGGAGTCAACGGATTAGACCAGTGACTCTGACTGCT |
|  |  |  | Allele 2 | GAAGGTGACCAAGTTCATGCTACCAGTGACTCTGACTGCG |
|  |  |  | Reverse | GAGAGAACAGGGCAAAGACAAA |
|  | 143,274,837 (SNP 6) | T/C | Allele 1 | GAAGGTCGGAGTCAACGGATTAGAGGGAAGAAGGCTGGGG |
|  |  |  | Allele 2 | GAAGGTGACCAAGTTCATGCTAAAGAGGGAAGAAGGCTGGGA |
|  |  |  | Reverse | ATGAGGGAGTCTAAAGGGCT |
|  | 143,274,943 (SNP 7) | C/G | Allele 1 | GAAGGTCGGAGTCAACGGATTACGTAGATCTTTGTACTCCTGC |
|  |  |  | Allele 2 | GAAGGTGACCAAGTTCATGCTAACGTAGATCTTTGTACTCCTGG |
|  |  |  | Reverse | GCACCAACAAGAAAGGGGAAT |
| *Slc22a18* | 143,303,959 | T/C | Allele 1 | GAAGGTCGGAGTCAACGGATTCCATACCTTAGGACTCG |
|  |  |  | Allele 2 | GAAGGTGACCAAGTTCATGCTGATTCCATACCTTAGGACTCA |
|  |  |  | Reverse | GCTCTGGGGAGTGGCATA |
|  | 143,304,188 | C/T | Allele 1 | GAAGGTCGGAGTCAACGGATTTCCCAAGTGCTGGGATTAAAGAC |
|  |  |  | Allele 2 | GAAGGTGACCAAGTTCATGCTTCTCCCAAGTGCTGGGATTAAAGAT |
|  |  |  | Reverse | GTCTCTCTTGAGGGGTCCTTAA |
|  | 143,304,441 | C/T | Allele 1 | GAAGGTCGGAGTCAACGGATTGGAGTGGATGATGGATGGGTTAGG |
|  |  |  | Allele 2 | GAAGGTGACCAAGTTCATGCTGGAGTGGATGATGGATGGGTTAGA |
|  |  |  | Reverse | GGTCCTCCCAGGGTCTTT |
|  | 143,304,455 | C/A | Allele 1 | GAAGGTGACCAAGTTCATGCTTTGTCCCCATGGAGAT |
|  |  |  | Allele 2 | GAAGGTCGGAGTCAACGGATTTTGTCCCCATGGAGCT |
|  |  |  | Reverse | AGGAGTGGATGATGGATGGGTT |
| *Nap1l4* | 143,373,324 | G/A | Allele 1 | GAAGGTCGGAGTCAACGGATTCCTACCTGCTCTGTTGGTT |
|  |  |  | Allele 2 | GAAGGTGACCAAGTTCATGCTATTCCTACCTGCTCTGTTGGC |
|  |  |  | Reverse | CAGGAGACAGGGATGCTGAA |
|  | 143,373,708 | G/T | Allele 1 | GAAGGTCGGAGTCAACGGATTGTTTATACGCCTACACACCACG |
|  |  |  | Allele 2 | GAAGGTGACCAAGTTCATGCTGGTTTATACGCCTACACACCACT |
|  |  |  | Reverse | CGAAAGATTGCCCGAGAGAAAA |

Supplemental Table 2: Additional SNP markers and their corresponding Amplifluor Assay oligos for fine mapping the *Kcnq1*, *Cdkn1c*, *Slc22a18* and *Nap1l4* hotspots within *Kcnq1* transcriptionally imprinted domain.
